# Selection of Reference Genes for Normalization of MicroRNA Expression by RT-qPCR in Sugarcane Buds under Cold Stress

**DOI:** 10.3389/fpls.2016.00086

**Published:** 2016-02-05

**Authors:** Yuting Yang, Xu Zhang, Yun Chen, Jinlong Guo, Hui Ling, Shiwu Gao, Yachun Su, Youxiong Que, Liping Xu

**Affiliations:** Key Laboratory of Sugarcane Biology and Genetic Breeding Ministry of Agriculture, Fujian Agriculture and Forestry UniversityFuzhou, China

**Keywords:** sugarcane, miRNAs, RT-qPCR, reference gene, cold stress

## Abstract

Sugarcane, accounting for 80% of world's sugar, originates in the tropics but is cultivated mainly in the subtropics. Therefore, chilling injury frequently occurs and results in serious losses. Recent studies in various plant species have established microRNAs as key elements in the post-transcriptional regulation of response to biotic and abiotic stresses including cold stress. Though, its accuracy is largely influenced by the use of reference gene for normalization, quantitative PCR is undoubtedly a popular method used for identification of microRNAs. For identifying the most suitable reference genes for normalizing miRNAs expression in sugarcane under cold stress, 13 candidates among 17 were investigated using four algorithms: geNorm, NormFinder, deltaCt, and Bestkeeper, and four candidates were excluded because of unsatisfactory efficiency and specificity. Verification was carried out using cold-related genes *miR319* and *miR393* in cold-tolerant and sensitive cultivars. The results suggested that *miR171*/*18S rRNA* and *miR171*/*miR5059* were the best reference gene sets for normalization for miRNA RT-qPCR, followed by the single *miR171* and *18S rRNA*. These results can aid research on miRNA responses during sugarcane stress, and the development of sugarcane tolerant to cold stress. This study is the first report concerning the reference gene selection of miRNA RT-qPCR in sugarcane.

## Introduction

In recent years, microRNAs (miRNAs) have received increasing attention for their vital roles in the post-transcriptional or translational regulation of gene expression. MiRNAs, as one class of small non-coding RNAs, are 18- to 25-nucleotides that exist in most eukaryotic genomes. miRNAs, which are excised from the primary miRNA by dicer-like1 enzymes (DCL1, RNase III enzymes), can silence the expression of target RNAs containing their complementary sequences in plants by binding and guiding their effector proteins (Rogers and Chen, 2013), and thus play key roles by negatively regulating target genes in many biological processes, including growth, development, hormone signal transduction, and response to various stresses (Bartel, 2004; Jin, 2008; Padmanabhan et al., 2009; Sunkar, 2010; Sunkar et al., 2012). At first, the methods mainly used to detect the miRNA included microarray and Northern blot (Chen et al., 2005; Davoren et al., 2008). As the mature miRNAs have small size and sequence similarity, it was a great challenge to apply reverse transcription quantitative real-time polymerase chain reaction (RT-qPCR) to detect miRNAs, until stem loop RT-qPCR and poly(A)-tailed RT-qPCR approaches have been developed to characterize mature miRNAs (Chen et al., 2005; Shi and Chiang, 2005).

RT-qPCR, is commonly used to target and quantify gene expression. Recently, RT-qPCR has been used to investigate the expression of miRNAs to better understand their function. The poly (A)-tailed RT-qPCR approach for quantifying miRNAs uses poly-adenlyation, elongation, and reverse transcription in separated steps. The stem loop RT-qPCR method developed by Chen et al. (2005) uses steps that take mature miRNA which are specifically converted into DNA complements by stem loop primers, followed by the quantitative real-time polymerase chain reaction. Compared with the poly (A)-tailed RT-qPCR approach, the stem loop RT-qPCR method makes it possible to enable more specific and sensitive PCR-based miRNA quantification.

Although, the RT-qPCR is widely applied to quantification of biologically relevant changes in mRNA and miRNA levels, the accuracy of RT-qPCR is easily influenced by cDNA quality, simple amount, RNA integrity and efficiencies of the reverse transcription (RT) and PCR. The normalization step is a key pre-condition and several strategies are used to normalize the RT-qPCR data. Among these, the application of one or several reference genes is an effective way to balance the variation between samples and reactions (Vandesompele et al., 2002; Nicot et al., 2005; Ovesná et al., 2012). A perfect reference gene is supposed to be expressed stably and constantly under any condition, such as at different development stages or under various abiotic and biotic stresses. Besides, its expression level cannot be influenced by experimental parameters (Thellin et al., 1999; Schmittgen and Zakrajsek, 2000). In addition, the reference gene should have a similar expression level with the target gene in the samples being analyzed (Cappelli et al., 2008). According to previous studies, various reference genes for miRNA RT-qPCR have been used in plants, including the 5S ribosomal RNA (*5S rRNA*), 5.8S ribosomal RNA (*5.8S rRNA*), U6 small nuclear RNA (*U6 snRNA*), and glyceraldehyde-3-phosphate dehydrogenase (*GAPDH*; Song et al., 2010; Yu et al., 2011; Gao et al., 2012; Thiebaut et al., 2012). To date, only a few reports are related to the selection of reference genes for miRNA RT-qPCR, such as in *Amygdalus persica* L. (Luo et al., 2014), *Triticum aestivum* L. (Feng et al., 2012), *Dimocarpus longan* Lour. (Lin and Lai, 2013), *Citrus reticulata* Blanco. (Kou et al., 2012), and *Glycine max* L. (Kulcheski et al., 2010). Several studies on the miRNA in sugarcane (*Saccharum* spp.) have also been reported, while they all focused on the identification of miRNAs by high-throughput sequencing method or functional verification of miRNAs in sugarcane (Ferreira et al., 2012; Thiebaut et al., 2012; Bottino et al., 2013). Till now, there is no report on reference gene evaluation for miRNA RT-qPCR in sugarcane.

Sugarcane, with a total of 19.4 million hectares (Mha) of world production (Pinto et al., 2005), is the major sugar crop, as well as a promising industrial raw material for biofuel and some food industries, such as rum, and its cultivation area is expanding. In 2012, it was cultivated in 101 countries, on ~26.1 Mha and accounted for 80% of all sugar produced in the world and 92% in China according to the data from FAO (Que et al., 2014). However, due to its origin in the tropics and cultivation in subtropical regions, sugarcane has low cold tolerance, usually leading to a serious yield loss when exposed to low temperatures (Li and Yang, 2015; Zhang et al., 2015). For example, low temperatures from January to February 2008 in China, resulted in serious yield losses and the total sugar produced decreased from 1.484 million ton (Mt) in the 2007/2008 to 1.243 Mt in the 2008/2009 crop season. The damage was extended due to the injury of bud which resulted in the germination rate decreasing in the ratoon crop and in the next plant cane. In addition, chilling damage has occurred since 2008 in China. The ratoon, regrowth of previously planted cane, is crucial for the sugarcane production and prolonged ratoon time can save the cost and benefit to the environment, thus one plant cane and three to four ratoon is normally adopted in the sugarcane planting system. When suffering from exposure to cold, the buds turn brown, vitality decreases, and some buds die, leading to the ratoon with not enough seedlings per acre, and poor growth of seedlings that do survive. In addition, low temperature is conducive to sucrose accumulation during mature period (Ebrahim et al., 1998) and thus, the harvest normally begins in winter and continues to spring. For plant cane, sowing season coincides with cold in most cane cultivation regions, therefore cold tolerance in bud is critical. For these reasons our study focused on the cold stress of sugarcane buds.

Recently, the study of miRNA regulation has shown that it has a key role in cold-related molecular mechanisms (Sunkar et al., 2012; Thiebaut et al., 2012). Several experiments have been carried out to discover and identify the cold-related miRNAs on miRNAs sequencing data in sugarcane cultivars FN39 (cold tolerant) and ROC22 (cold sensitive) challenged with low (4°C) temperature for 0 h (before the cold treatment), 3 h and 12 h (unpublished). At present, although RT-qPCR is a very common method to detect miRNAs, there is no reference for us to choose the appropriate gene for normalization. In this study, to normalize sugarcane miRNA expression during cold stress, after removing the four candidates with unsatisfactory efficiency and specificity, *miR156, miR396, U6 snRNA* (Accession number: CA294483), and 25S ribosomal RNA (2*5S rRNA*, Accession number: BQ536525), 13 candidate reference genes, including 9 miRNAs, *5S rRNA* (Accession number: HS075897), 18S ribosomal RNA (*18S rRNA*, Accession number: SCFRRE06), *GAPDH* (Accession number: CA254672) and eukaryotic elongation factor 1-alpha (*eEF-1*α, Accession number: EF581011.1), were evaluated by stem loop RT-qPCR method using four statistical algorithms: geNorm (Vandesompele et al., 2002), NormFinder (Andersen et al., 2004), deltaCt (Silver et al., 2006), and Bestkeeper (Pfaffl et al., 2004). The aim was to select suitable reference genes which can express stably in sugarcane buds under both normal condition and cold stress, and could thus, serve as normalization factors for miRNA RT-qPCR experimental analysis.

## Results

### Amplification characteristics of candidate reference genes

A total of 17 candidate reference genes, including 11 miRNAs, 2 small RNAs, and 4 housekeeping genes, were selected for miRNA expression normalization in sugarcane samples. Among them, the expression level of 11 miRNAs, selected from our small RNA library by high-throughput sequencing, had little difference between the cold-treated samples and the control through differential expression analysis (|log_2_(Treatment/Control)| < 1), and their sequences information were shown in Table 1. The melting curves showed the specificity of primers (Supplementary Figure 1). The *E* and *R*^2^-values refer to the efficiency of the reaction and the matching degree of the plotted data points to the standard curve in the PCR, respectively (Feng et al., 2012). Good primers should meet the following criteria: the standard curve generated is linear, the *R*^2^-value is more than 0.98, and the *E*-value tends to 100%. When the *E*-value approaches 100%, the target cDNA is duplicated in each PCR cycle of the exponential phase (Kulcheski et al., 2010). In this test, the efficiency values of the 17 candidate genes ranged from 0.72 to 1.85 (Table 2). According to the criterion mentioned above, four genes including *miR156, miR396, U6 snRNA*, and *25S rRNA* were excluded due to unsatisfactory efficiency and specificity. The remaining 13 candidates, with *E*-values ranging from 0.81 to 1.21 (Luo et al., 2014), and the *R*^2^-values higher than 0.98, were kept for further investigation.

**Table 1:** **The sequences and stem loop primers of the 11 candidate reference miRNAs**.

**Gene**	**Sequence**	**Stem loop primer**
*miR156*	TGACAGAAGAGAGTGAGCAC	GTCGTATCCAGTGCAGGGTCCGAGGTATTCGCACTGGATACGACGTGCTCAC
*miR159*	TTTGGATTGAAGGGAGCTCTG	GTCGTATCCAGTGCAGGGTCCGAGGTATTCGCACTGGATACGACCAGAGCTC
*miR160*	GCGTGCAAGGAGCCAAGCATG	GTCGTATCCAGTGCAGGGTCCGAGGTATTCGCACTGGATACGACCATGCTTG
*miR167*	TGAAGCTGCCAGCATGATCTGA	GTCGTATCCAGTGCAGGGTCCGAGGTATTCGCACTGGATACGACTCAGATCA
*miR171*	TGATTGAGCCGTGCCAATATC	GTCGTATCCAGTGCAGGGTCCGAGGTATTCGCACTGGATACGACGATATTGG
*miR396*	GGTCAAGAAAGCTGTGGGAAG	GTCGTATCCAGTGCAGGGTCCGAGGTATTCGCACTGGATACGACCTTCCCAC
*miR398*	GCAGGTGATGAGAACAAGA	GTCGTATCCAGTGCAGGGTCCGAGGTATTCGCACTGGATACGACTCTTGTTC
*miR1520*	ATCAGAACTGGTACGGACAA	GTCGTATCCAGTGCAGGGTCCGAGGTATTCGCACTGGATACGACTTGTCCGT
*miR5059*	CGTTCCTGGGCAGCAACACCA	GTCGTATCCAGTGCAGGGTCCGAGGTATTCGCACTGGATACGACTGGTGTTG
*miR5072*	GTTCCCCAGCGGAGTCGCCA	GTCGTATCCAGTGCAGGGTCCGAGGTATTCGCACTGGATACGACTGGCGACT
*miR5655*	AACACATGTGGATTGAGATGGATA	GTCGTATCCAGTGCAGGGTCCGAGGTATTCGCACTGGATACGACTATCCATC

**Table 2:** **Primer sequences and amplicon characteristics for each of the 17 candidate reference genes**.

**Gene**	**Forward primer (5′–3′)**	**Reverse primer (5′–3′)**	**PCR efficiency (*E*%)**	**Regression coefficient (*R*^2^)**	**Mean *Ct***	***SD***	***CV* (%)**
*miR159*	AGCGGTTTGGATTGAAGGGA	GTGCAGGGTCCGAGGT	0.95	0.990	20.58	0.7221	3.51
*miR160*	ATTATGCGTGCAAGGAGCCA	GTGCAGGGTCCGAGGT	1.05	0.994	25.23	0.5711	2.26
*miR167*	ATCGTGAAGCTGCCAGCATG	GTGCAGGGTCCGAGGT	1.03	0.998	21.37	0.6239	2.92
*miR171*	ATACGTGATTGAGCCGTGCC	GTGCAGGGTCCGAGGT	0.99	0.999	20.38	0.4944	2.43
*miR398*	ATTCGCCGCAGGTGATGAGA	GTGCAGGGTCCGAGGT	1.05	0.997	24.69	0.5285	2.14
*miR1520*	CGGCGGATCAGAACTGGTAC	GTGCAGGGTCCGAGGT	1.21	0.994	17.44	0.8434	4.84
*miR5059*	ATCATCGTTCCTGGGCAGCA	GTGCAGGGTCCGAGGT	1.00	0.997	20.53	0.6742	3.28
*miR5072*	ATATGAGTTCCCCAGCGGAG	GTGCAGGGTCCGAGGT	1.03	0.999	17.03	0.5578	3.27
*miR5655*	CGGCAACACATGTGGATTGAGA	GTGCAGGGTCCGAGGT	1.06	0.999	28.15	0.6071	2.16
*5S rRNA*	GCGTAGAGGAACCACACCAATC	CGAGCTATTTTGCCGCAGG	1.09	0.999	18.94	0.5799	3.06
*18S rRNA*	CTACGTCCCTGCCCTTTGTACA	ACACTTCACCGGACCATTCAA	0.98	0.982	14.51	0.3093	2.13
*GAPDH*	CACGGCCACTGGAAGCA	TCCTCAGGGTTCCTGATGCC	0.83	0.988	21.94	0.8473	3.86
*eEF-1α*	TTTCACACTTGGAGTGAAGCAGAT	GACTTCCTTCACAATCTCATCATAA	0.84	0.995	21.93	0.6636	3.02
*miR156*	AGGCGCCTGACAGAAGAGAGT	GTGCAGGGTCCGAGGT	0.77	0.978	–	–	–
*miR396*	ACCTGCGGTCAAGAAAGCTGT	GTGCAGGGTCCGAGGT	1.85	0.932	–	–	–
*U6 snRNA*	ACAGAGAAGATTAGCATGGCCC	GACCATTTCTCGATTTATGCGTG	0.72	0.966	–	–	–
*25S rRNA*	GCAGCCAAGCGTTCATAGC	CCTATGGTGGGTGAACAATCC	1.26	0.921	–	–	–

As shown in Table 2, the mean *Ct*-values of the 13 candidates from all the 14 sugarcane samples (buds with 0, 1, 3, 6, 12, 24, 48 h cold-treatment of FN39 and ROC22 cultivars) varied from 14.51 to 28.15. The co-variance (*CV*) values ranged from 2.13 to 4.84%. On the basis of mean *Ct*, the *18S rRNA* had the highest expression level among the 13 genes with the lowest mean *Ct*-value (14.51), while the *miR5655* showed the least expression with the highest mean *Ct*-value (28.15). On the whole, the ranking of gene expression level by *Ct*-values was *18S rRNA* >*miR5072* >*miR1520* >*5SrRNA* >*miR171* >*miR5059* >*miR159* >*miR167* >*eEF-1*α >*GAPDH* >*miR398* >*miR160* >*miR5655*. Furthermore, the *18S rRNA* had the least variability with a *CV*-value of 2.13% among 13 candidate reference genes, while *miR1520* was the least stable gene with a *CV*-value of 4.84%. The other 11 candidate genes varied from 2.14 to 3.86%. According to the *CV*-values, the whole rank of gene stability was *18S rRNA* >*miR398* >*miR5655* >*miR160* >*miR171* >*miR167* >*eEF-1*α >*5SrRNA* >*miR5072* >*miR5059* >*miR159* >*GAPDH* >*miR1520*.

### Expression stability of candidate reference genes

The expression stability of 13 candidate genes in FN39 and ROC22 cultivars under cold stress was evaluated by geNorm, NormFinder, Bestkeeper, and deltaCt method. The stability values (SV) were used to rank these genes from the most to the least stable (Tables 3–5). In addition, the mean *Ct*-values of 13 genes in each sample were shown in Supplementary Table 1.

**Table 3:** **Relative expression stability of the 13 candidate reference genes in the buds of sugarcane FN39 cultivar under cold stress**.

**Rank**	**geNorm**			**NormFinder**			**deltaCt**			**Bestkeeper**		
	**Gene**	***SV***	***SE***	**Gene**	***SV***	***SE***	**Gene**	***SV***	***SE***	**Gene**	***SV***	***SE***
1	*miR171*	0.005	0.023	*miR5059*	0.003	0.034	*miR167*	1.444	0.014	*18S rRNA*	0.162	0.005
2	*18S rRNA*	0.005	0.031	*miR167*	0.004	0.021	*18S rRNA*	1.457	0.034	*miR167*	0.235	0016
3	*miR5059*	0.009	0.009	*miR171*	0.009	0.026	*5S rRNA*	1.461	0.022	*5S rRNA*	0.253	0.013
4	*miR167*	0.012	0.012	*18S rRNA*	0.010	0.037	*eEF-1α*	1.505	0.015	*miR171*	0.327	0.031
5	*miR398*	0.021	0.022	*miR398*	0.012	0.051	*miR171*	1.545	0.054	*eEF-1α*	0.378	0.023
6	*5S rRNA*	0.027	0.034	*miR5655*	0.019	0.054	*GAPDH*	1.618	0.061	*miR398*	0.380	0.014
7	*miR160*	0.031	0.043	*5S rRNA*	0.021	0.029	*miR398*	1.632	0.023	*GAPDH*	0.408	0.045
8	*miR159*	0.036	0.055	*miR160*	0.023	0.061	*miR5655*	1.647	0.022	*miR160*	0.433	0.025
9	*miR5655*	0.041	0.041	*miR159*	0.038	0.055	*miR160*	1.674	0.045	*miR5655*	0.450	0.032
10	*eEF-1α*	0.057	0.031	*eEF-1α*	0.085	0.031	*miR159*	1.676	0.007	*miR159*	0.450	0.024
11	*GAPDH*	0.072	0.025	*GAPDH*	0.099	0.019	*miR1520*	1.838	0.014	*miR1520*	0.609	0.044
12	*miR5072*	0.102	0.014	*miR5072*	0.172	0.017	*miR5072*	5.412	0.027	*miR5072*	3.785	0.022
13	*miR1520*	0.128	0.064	*miR1520*	0.180	0.021	*miR5059*	6.410	0.011	*miR5059*	4.498	0.031

*The SV, stability value. The SE, standard error*.

**Table 4:** **Relative expression stability of the 13 candidate reference genes in the buds of sugarcane ROC22 cultivar under cold stress**.

**Rank**	**geNorm**			**NormFinder**			**deltaCt**			**Bestkeeper**		
	**Gene**	***SV***	***SE***	**Gene**	***SV***	***SE***	**Gene**	***SV***	***SE***	**Gene**	***SV***	***SE***
1	*miR171*	0.002	0.011	*miR171*	0.001	0.022	*miR398*	0.324	0.021	*18S rRNA*	0.098	0.046
2	*miR5059*	0.002	0.016	*18S rRNA*	0.001	0.015	*miR171*	0.331	0.009	*5S rRNA*	0.326	0.061
3	*18S rRNA*	0.003	0.009	*miR5059*	0.001	0.016	*miR160*	0.344	0.005	*miR5655*	0.349	0.024
4	*miR167*	0.016	0.005	*miR167*	0.003	0.043	*miR167*	0.370	0.045	*GAPDH*	0.377	0.031
5	*miR5072*	0.020	0.012	*miR5072*	0.003	0.061	*miR5655*	0.372	0.050	*miR171*	0.386	0.025
6	*miR160*	0.023	0.032	*miR160*	0.009	0.031	*miR1520*	0.384	0.027	*miR160*	0.404	0.022
7	*miR398*	0.024	0.056	*miR398*	0.015	0.034	*miR159*	0.409	0.020	*miR398*	0.416	0.030
8	*miR5655*	0.026	0.034	*miR5655*	0.024	0.041	*5S rRNA*	0.423	0.043	*miR1520*	0.473	0.016
9	*5S rRNA*	0.031	0.061	*5S rRNA*	0.036	0.022	*miR5072*	0.462	0.033	*eEF-1α*	0.474	0.018
10	*miR159*	0.039	0.025	*miR159*	0.048	0.026	*GAPDH*	0.515	0.010	*miR167*	0.476	0.045
11	*GAPDH*	0.058	0.047	*GAPDH*	0.094	0.016	*18S rRNA*	0.528	0.017	*miR5072*	0.489	0.054
12	*eEF-1α*	0.073	0.038	*eEF-1α*	0.108	0.015	*eEF-1α*	0.528	0.006	*miR159*	0.532	0.026
13	*miR1520*	0.091	0.053	*miR1520*	0.126	0.007	*miR5059*	0.587	0.013	*miR5059*	0.745	0.065

*The SV, stability value. The SE, standard error*.

**Table 5:** **Relative expression stability of the 13 candidate reference genes in the buds of sugarcane FN39 and ROC22 cultivars under cold stress**.

**Rank**	**geNorm**			**NormFinder**			**deltaCt**			**Bestkeeper**		
	**Gene**	***SV***	***SE***	**Gene**	***SV***	***SE***	**Gene**	***SV***	***SE***	**Gene**	***SV***	***SE***
1	*miR171*	0.004	0.015	*miR171*	0.001	0.046	*miR167*	1.185	0.032	*18S rRNA*	0.259	0.004
2	*18S rRNA*	0.004	0.022	*18S rRNA*	0.001	0.012	*5S rRNA*	1.197	0.015	*miR171*	0.365	0.021
3	*miR5059*	0.007	0.016	*miR398*	0.003	0.009	*18S rRNA*	1.199	0.044	*miR398*	0.421	0.034
4	*miR167*	0.016	0.033	*miR5059*	0.003	0.051	*miR171*	1.203	0.054	*miR5655*	0.431	0.033
5	*miR160*	0.024	0.023	*miR167*	0.008	0.024	*eEF-1α*	1.238	0.013	*miR160*	0.482	0.040
6	*miR398*	0.028	0.051	*miR160*	0.017	0.065	*miR5655*	1.244	0.024	*5S rRNA*	0.490	0.029
7	*miR5655*	0.033	0.053	*miR5655*	0.020	0.052	*GAPDH*	1.289	0.017	*miR167*	0.493	0.061
8	*5S rRNA*	0.036	0.024	*5S rRNA*	0.029	0.034	*miR159*	1.313	0.037	*GAPDH*	0.518	0.055
9	*miR159*	0.042	0.028	*miR159*	0.041	0.044	*miR398*	1.337	0.029	*eEF-1α*	0.537	0.040
10	*GAPDH*	0.062	0.034	*GAPDH*	0.093	0.063	*miR160*	1.413	0.058	*miR1520*	0.555	0.027
11	*eEF-1α*	0.075	0.032	*eEF-1α*	0.096	0.023	*miR1520*	1.540	0.053	*miR159*	0.595	0.064
12	*miR5072*	0.094	0.045	*miR5072*	0.123	0.016	*miR5072*	3.792	0.046	*miR5072*	2.084	0.045
13	*miR1520*	0.115	0.014	*miR1520*	0.155	0.044	*miR5059*	4.506	0.043	*miR5059*	2.449	0.033

*The SV, stability value. The SE, standard error*.

According to the geNorm software instruction, the geNorm calculates the gene expression stability measure *M* for a reference gene as the average pairwise variation for that gene with all other tested reference genes. Stepwise exclusion of the gene with the highest *M*-value allows ranking of the candidate reference genes according to their expression stability. The genes with the highest or lowest *M*-value had the least or most stability of expression, respectively (Vandesompele et al., 2002; Kulcheski et al., 2010). The program NormFinder uses a mathematical model of gene expression, which can carry out an estimation on the inter- and intra- group variation (Andersen et al., 2004). Similar to the geNorm program, the smaller the stability value, the more stable the gene is. The strategy of the deltaCt method is to compare relative expression of “pairs of genes” in each sample to confidently identify useful reference genes (Silver et al., 2006). The Bestkeeper software not only determines the optimal reference gene by employing the pair-wise correlation analysis of all candidate pairs, but also calculates the geometric average of the best suited ones (Pfaffl et al., 2004). In the cold-tolerant cultivar FN39, the *miR171, 18S rRNA, miR167* ranked among the top five candidate genes from geNorm, NormFinder, Bestkeeper, or deltaCt under cold stress. The genes *miR398, miR5059, 5S rRNA*, and *eEF-1*α had stable expression in two systematic statistical algorithms. In the cold-sensitive sugarcane cultivar ROC22, *miR171* was the most stable as in the FN39, and the *18S rRNA, miR167, miR5655, miR5059*, and *miR5072* were evaluated as more stable genes in at least two algorithms. The genes *miR171* and *18S rRNA* were still ranked in the top five when evaluated by all four algorithms in both cultivars. *MiR167* also showed high expression stability using the geNorm, NormFinder, and deltaCt programs. Taken together, the expression patterns of *miR171, 18S rRNA*, and *miR167* were more stable than the other 10 candidate genes, indicating that they may be suitable as the reference genes in RT-qPCR.

In this study, the Pearson correlation analysis was applied to evaluate the uniformity of candidate reference genes ranks obtained by geNorm, NormFinder, Bestkeeper, or deltaCt programs (Table 6). In both cultivars, the Pearson correlation coefficients (*r*) between two methods of geNorm and NormFinder, geNorm and Bestkeeper, and NormFinder and Bestkeeper were significant, which meant that the evaluation results based on the pair methods were similar. The results based on deltaCt method had less correlation with those of the other three program analysis. The highest correlation coefficients of the ranks of all candidate reference gene sets by two methods was geNorm and NormFinder due to the *r*-values in FN39, ROC22, and FN39+ROC22 were 0.919, 0.873, and 0.913, respectively (Table 6).

**Table 6:** **The correlation analysis of the 13 candidate reference genes ranks based on the evaluation of four statistical algorithms**.

**Algorithm**	**Correlation coefficient**		
	**FN39**	**ROC22**	**FN39+ROC22**
geNorm vs. NormFinder	0.919^**^	0.873^**^	0.913^**^
geNorm vs. deltaCt	0.507	0.653^*^	0.640^*^
geNorm vs. Bestkeeper	0.790^**^	0.565	0.810^**^
NormFinder vs. deltaCt	0.637^*^	0.541	0.646^*^
NormFinder vs. Bestkeeper	0.813^**^	0.862^**^	0.911^**^
deltaCt vs. Bestkeeper	0.505	0.691^*^	0.656^*^

* *correlation is significant at p < 0.05*,

** *correlation is significant at p < 0.01*.

### Use of multiple reference genes

It has been recommended that normalization methods using multiple genes measure expression level accurately (Vandesompele et al., 2002). To test this, the normalization factor (*NF*) and the pairwise variation (*V*) were calculated by the geNorm to determine the optimal number of reference genes (Vandesompele et al., 2002). By setting a threshold of *V* = 0.15, the optimal gene number was evaluated by the geNorm for reliable normalization (Vandesompele et al., 2002).

**Figure 1:**
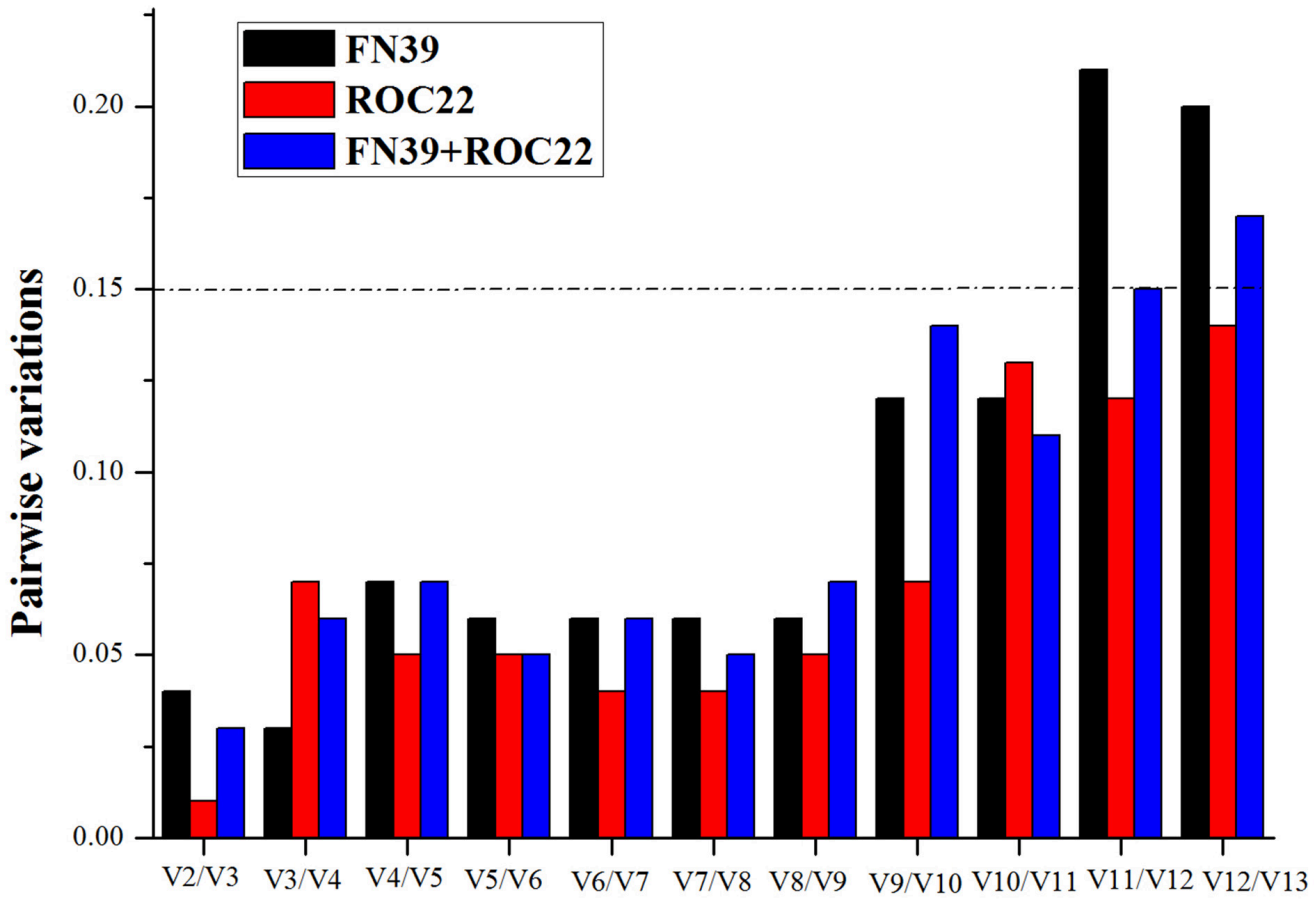
**Determination of the optimal number of reference genes in the buds of sugarcane FN39 and ROC22 cultivars under cold stress**. NFn was the Normalization Factor which was based on n reference genes included in the present study. The pairwise variation was analyzed by the geNorm to determine the optimal number. The 0.15 was set as a threshold value, suggesting it was not necessary to add one or more genes into the combination of reference genes. As shown in Figure 1, the optimal number of reference genes under cold stress in FN39, ROC22, and FN39+ROC22 were all two, suggesting that the top two genes ranked by geNorm were the best combination of reference genes for normalization in miRNA RT-qPCR.

In order to obtain the optimal number of reference genes, the pairwise variations for all the data were calculated (Figure 1). According to the results, irrespective of cultivar, the V2/V3 is lower than 0.15, indicating the two most stable genes were sufficient for normalization. The addition of a third, fourth, or fifth gene did not cause a significant decrease of the *V*-value (Vandesompele et al., 2002). Therefore, *miR171, 18S rRNA, miR167, miR171*/*18S rRNA*, and *miR171*/*miR5059* were analyzed, as they were the best single reference genes and the optimal gene sets evaluated under cold stress in two sugarcane cultivar buds. Furthermore, the *5S rRNA* and *GAPDH* were also used to normalize the expression level of the two target genes, since they were often used as reference genes for miRNA expression in previous studies. Meanwhile, the combination of *miR171, 18S rRNA*, and *miR5059* was also set as the reference.

### Validation of putative reference genes

**Figure 2:**
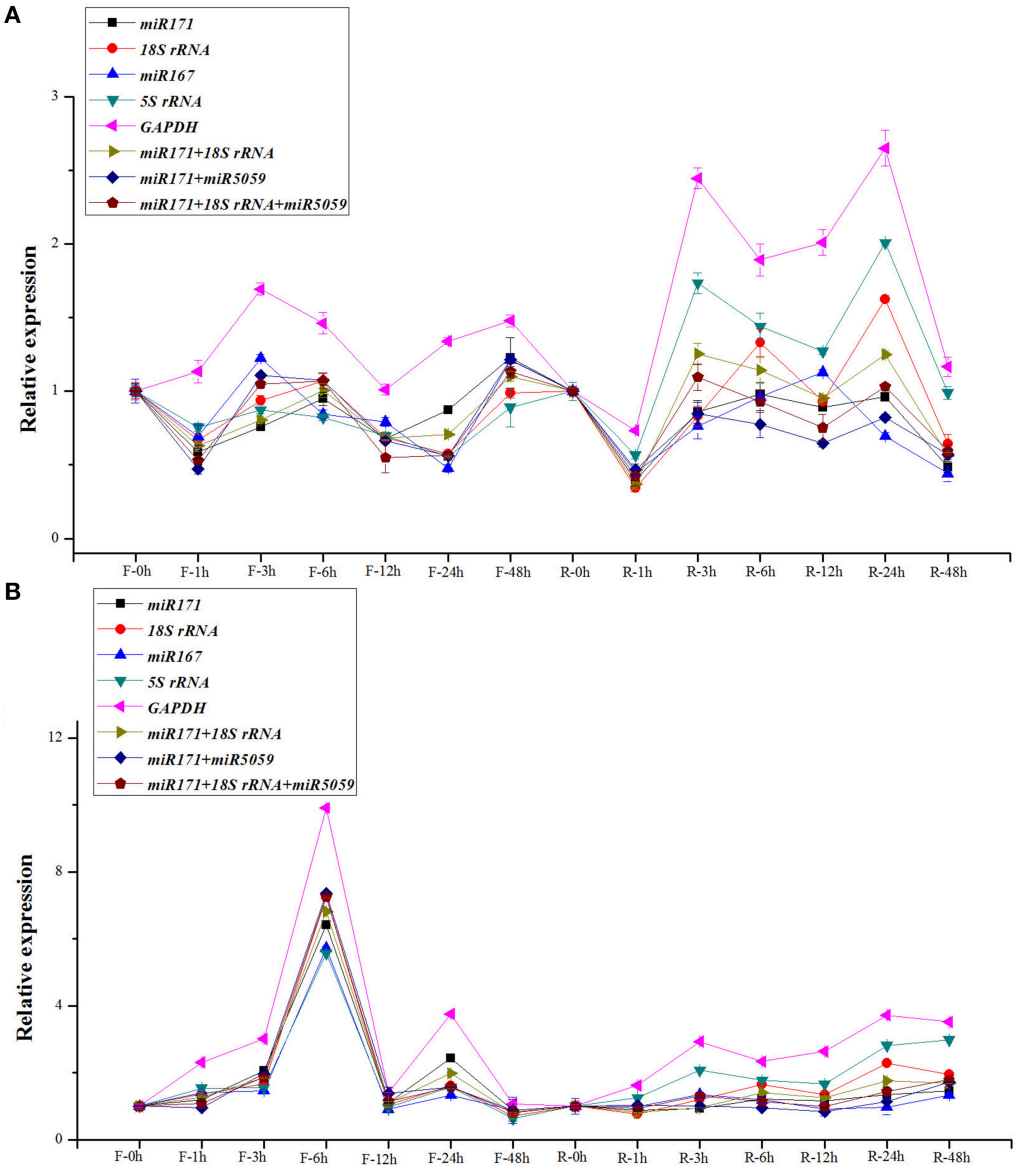
**Normalization expression of *miR319* and *miR393* in the buds of sugarcane FN39 and ROC22 cultivars under cold stress**. In this study, the normalization of *miR319* **(A)** and *miR393* **(B)** employed the single reference *miR171, 18S rRNA, miR167, 5S rRNA, GAPDH*, and gene sets *miR171*/*18SrRNA, miR171*/*18S rRNA, miR171*/*18S rRNA*/*miR5059* in FN39 and ROC22 cultivars. F meant the FN39 cultivar, and R meant the ROC22 cultivar.

In this study, the *miR319* and *miR393*, which were both reported as cold-related miRNAs in different plant species (Zhang et al., 2009; Thiebaut et al., 2012; Wang et al., 2014), were selected as the target genes to validate the above selected reference genes by RT-qPCR under cold stress in the two sugarcane cultivars. Based on the results derived from the samples collected in all the time points of FN39 and ROC22 cultivars, the expression patterns of *miR319* were similar (Figure 2A). According to our previous high-throughput sequencing results (unpublished), the expression of *miR319* has a slight down-regulation at 3 and 12 h in FN39 and ROC22 cultivars with cold treatment. In FN39, the expression level of *mi319* with *miR171, 18S rRNA, 5S rRNA*, or *miR171/18S rRNA* normalization at 3 h was down-regulated. Expression also dropped slightly at 12 h when normalized by all the candidate reference genes except the *GAPDH*. Therefore, when *miR171, 18S rRNA, 5S rRNA*, and *miR171/18S rRNA* were used as reference genes respectively, the *miR319* expression level was similar with the previous high-throughput sequencing study in FN39. In ROC22, the accumulation of *miR319* transcript decreased both at 3 h normalized by *miR171, 18S rRNA, miR167*, or *miR171/miR5059*, and at 12 h with the *miR171, 18S rRNA, miR171/miR5059, miR171/18S rRNA*, or *miR171/18S rRNA/miR5059* normalization. According to this validation, *miR319*, the *miR171, 18S rRNA*, and *miR171/18SrRNA* are the most suitable reference genes.

When normalized by *miR171, 18S rRNA, miR167, 5SrRNA, GAPDH, miR171*/*18S rRNA*, or *miR171*/*18S rRNA*/*miR5059*, the expression patterns of *miR393* showed a high similarity of the obviously up-regulation at 6 h in FN39 cold stress sample (Figure 2B). While in ROC22, the accumulation of the *miR393* transcript at each time point was not as consistent as that in FN39 when normalized by these candidate reference genes. Among them, the accumulation of the *miR319* transcript slightly decreased at 3 h when normalized by *miR171* or *miR171/18S rRNA* and increased at 12 h with *miR171, 18S rRNA, 5S rRNA, GAPDH*, or *miR171/18S rRNA* normalization. This change of *miR393* expression level at 3 and 12 h was identical with our previous high-throughput sequencing results (unpublished). From this point, *miR171* and *miR171/18S rRNA* are the most stable reference genes, followed by the single *18S rRNA*.

According to the pearson correction analysis (Table 7), the Pearson correlation coefficients (r) between RNA-Seq results and the RT-qPCR results with *miR171*/*18S rRNA, miR171*/*miR5059, miR171*, or *18S rRNA* normalization were all significant, indicating the expression patterns of *miR319* and *miR393* were similar. The results of the pearson analysis indirectly provided evidence that *miR171*/*18S rRNA, miR171*/*miR5059, miR171*, or *18S rRNA* were the better reference genes or gene combinations than the other candidate genes.

**Table 7:** **The Pearson correlation analysis of *miR319* and *miR393* expression profiles between the RT-qPCR results with different reference genes and the RNA-seq results in FN39 and ROC22**.

**RNA-Seq results**	**RT-qPCR results**							
	***miR171***	***18S rRNA***	***miR167***	***5S rRNA***	***GAPDH***	***miR171* + *miR5059***	***miR171* + *18S rRNA***	***miR171*+ *miR5059* + *18S rRNA***
*miR319*	0.632^**^	0.734^**^	0.321	0.493	0.302	0.628^**^	0.784^**^	0.632^*^
*miR393*	0.721^**^	0.608^**^	0.410	0.534^*^	0.374	0.541^*^	0.738^**^	0.432

* *correlation is significant at p < 0.05*,

** *correlation is significant at p < 0.01*.

## Discussion

Sugarcane is affected by several biotic and abiotic stresses that lead to decrease in cane yield and sucrose content. Cold stress is one constraint to sugarcane quality and productivity. In order to explore the molecular basis of cold stress, several methods have been used to measure gene expression, such as high-throughput sequencing, microarray, and RT-qPCR, among which the RT-qPCR is the most commonly used. There has been some research on the valuation of reliable reference genes in sugarcane (Ling et al., 2014), however without any report on the suitable reference gene selection for miRNA RT-qPCR analysis.

In the current study, 14 samples from two sugarcane cultivar buds under cold stress were collected to select the most suitable reference genes for RT-qPCR analysis. After the composite analysis using four algorithms (geNorm, NormFinder, Bestkeeper and deltaCt) and the validation of two miRNAs (*miR319* and *miR393*) responsive to the cold stress, the single genes *miR171*, and *18S rRNA*, and the gene sets *miR171*/*18S rRNA* and *miR171*/*miR5059* had the best performance among these candidate genes. In addition, some previous reports indicated that a single gene used as a reference gene for normalization was not as reliable as a gene set (Vandesompele et al., 2002; Janská et al., 2013). Based on the above analysis, the gene sets *miR171*/*18S rRNA* and *miR171*/*miR5059* were the best candidates for miRNA expression normalization under cold stress in sugarcane buds of FN39 and ROC22 cultivars, followed by single *miR171* and *18S rRNA*.

The gene *miR171* belongs to a conserved miRNA family, regulating the members of the *SCARECROW-LIKE* (*SCL*) transcription factor family. The *SCL* genes belong to the *GRAS* family, which play a part in gibberellic acid responses that regulate the development of apical meristem and flowering (Bolle, 2004; Lee et al., 2008). Studies in recent years have demonstrated *miR171* plays a key role in the differentiation of the meristem in many plants (Engstrom et al., 2011; Curaba et al., 2013). As the buds are one of the most important axillary meristems in sugarcane, it's reasonable to deduce that the expression of *miR171* should be relatively continuous and stable to promote the differentiation of the buds. This may explain why *miR171* has a relatively stable expression during the evaluation of reference genes in our study.

There are reports on the selection of *18S rRNA*, as a stable housekeeping gene, for use in RT-qPCR. Jain et al. (2006) found that the *18S rRNA* and *25S rRNA* were the most stable in *Oryza sativa* L. growth under various treatments, including hormone, drought and salt stress treatments. Yan et al. (2012) noted that the *18S rRNA*, beta actin *(ACTB)* and RNA polymerase II (*rp II*) were the most stable genes in the leaf of different citrus genotypes. In addition, Scholtz and Visser (2013) proved that the geometric mean of *18S rRNA* and beta tubulin (*TUBB*) showed the best stability in *Puccinia graminis* f.sp. *tritici*-infected wheat. In our current study, *18S rRNA* was also evaluated as the most stable single gene for normalization of miRNA RT-qPCR in sugarcane buds under cold stress. As is well-known, the ribosomes are the molecular machines to conduct the translation from nucleic acids to proteins in all organisms. Dependence of ribosome on rRNAs makes them conserved in both structure and sequence (Lagesen et al., 2007). Furthermore, Goidin et al. (2001) also believed that the level of rRNAs, constituting as much as 80% of the total RNA, was less likely to change under conditions that could lead to the variation of the mRNA expression. Together with the current knowledge, it makes sense to understand that the *18S rRNA* was evaluated as the stable reference gene for normalization of miRNA expression in RT-qPCR in cold-stressed sugarcane buds of two cultivars.

Ideally, a reference gene for quantitative gene expression studies should be stable and will not be affected by the experimental conditions, developmental stages or tissue types. However, in this study, *GAPDH* and *5S rRNA*, both often used as reference genes for gene expression under some environmental stresses, were not expressed in a stable pattern. This indicates the complexity of plant transcriptional events during response to the environment. Thus, evaluation should be undertaken to avoid the variation caused by the inappropriate reference genes.

In conclusion, the selection and validation of suitable reference genes for the normalization of miRNA RT-qPCR in sugarcane was achieved for the first time. Our study demonstrated that, for miRNA expression analysis in sugarcane under cold stress, *miR171*/*18S rRNA*, and *miR171*/*miR5059* were the best reference genes for multiple use, and the *miR171* and *18S rRNA* were the best single reference genes. These will be useful for studies on miRNA function in response to cold stress, and other abiotic stresses in sugarcane.

## Materials and methods

### Plant materials and stress treatment

The sugarcane stems of two non-transgenic disease-free sugarcane cultivars during technical maturing stage, the cold-sensitive ROC22 and the cold-tolerant FN39, were harvested from the field in the Key Laboratory of Sugarcane Biology and Genetic Breeding, Ministry of Agriculture (Fuzhou, China). The stems were cut to be one-bud node and treated firstly with flowing water for 2 days to promote the sprout of buds and remove impurities, then the nodes with buds were cultivated for sprouting (but without sprout) for 4 days by moisture culture at 28°C in darkness and 65% relative humidity condition in incubator (Dongqi, Ningbo, China). The consistent buds with 2–3 cm length from both cultivars were treated by exposure to low temperature (4°C) for 0 (before the cold treatment), 1, 3, 6, 12, 24, and 48 h. The whole process was carried out under darkness in incubator for simulating the natural condition. Each sample contained six buds and three biological replications were set at each time point. All samples were collected, quickly frozen in liquid nitrogen and then stored at –80°C before RNA extraction.

### RNA extraction and cDNA synthesis

For each sample, total RNA was extracted with the Trizol™ Reagent (Invitrogen, Carlsbad, CA, USA). The quality was evaluated by electrophoresis using a 1% agarose gel and measured by NanoVue Plus (GE healthcare, Little Chalfont, UK). The cDNA synthesis was carried out according to the instructions of TaqMan MicroRNA Reverse Transcription Kit (Applied Biosystems, Foster city, CA, USA). The reaction was performed at 16°C for 30 min, 42°C for 30 min, and 85°C for 5 min. All cDNA samples were 25-fold diluted before being used as a template in miRNA RT-qPCR analysis.

### Selection of candidate reference genes and primer design

In our previous study, the transcription levels of 11 conserved miRNAs were obtained and analyzed in sugarcane cultivar FN39 and ROC22 buds with the cold treatment by high-throughput sequencing (unpublished). The *5S rRNA* and *U6 snRNA* were the current frequently used reference genes of miRNA quantification in some plant species. For other genes, including *GAPDH, eEF-1*α, *18S rRNA*, and *25S rRNA*, were all chosen based on the previous report for sugarcane by Guo et al. (2014).

The stem loop primers used for miRNA cDNA synthesis were designed according to Chen et al. (2005). It contained a stem loop structure consisting of 44 conserved and 6 variable nucleotides which are specific to the 3′ end of the tested miRNA (Table 1). The forward primer in RT-qPCR was designed directly according to the sequence of target miRNA but without the six variable nucleotides at its 3′ end, while the universal reverse primer could be used for all mature miRNA (Table 2). The other primers were designed by the Primer 5.0 software following these rules: annealing temperature (Tm) of 60°C, amplified fragments length of 100–200 base-pairs, guanine-cytosine contents of 40–60%, and primers length of 17–25 nucleotides.

### RT-qPCR analysis

All the RT-qPCR was performed in the ABI 7500 Real-Time PCR system (Applied Biosystems, Foster city, CA, USA). The 20 μL reaction mix included 10.0 μL SYBR Green Master Mix (Roche, New York, New York, USA), 1.0 μM each primer, 2.0 μL 25-fold diluted cDNA and 6.0 μL ddH_2_O. The procedure was conducted for 2 min at 50°C, 10 min at 95°C, and followed for 40 cycles (15 s at 94°C and 60 s at 60°C), and melting curves were recorded immediately after the completion of the RT-qPCR to detect the specificity of the amplification. Three biological replications and three technological repeats were set in this study. The threshold cycle (*Ct*) values were collected for the further data analysis.

### Data analysis

A set of five 5-fold dilutions of cDNA from FN39 control samples were used to create the standard curves, and the correlation coefficient (*R*^2^) and PCR efficiency (E) were determined for each gene and each treatment using the linear regression model. The PCR efficiency was calculated following the formula E = 10^− 1∕slope^ − 1. For further in-depth analysis, the software programs of geNorm, NormFinder, Bestkeeper, and deltaCt were used to evaluate the stability of these candidate reference genes in the control samples and the cold-treated samples. For geNorm and NormFinder program analysis, the *Ct*-values were transformed into relative quantities following the formula: *Q* = *E*^▵*Ct*^. ▵*Ct* means the *Ct*-value difference between the treated sample and the control sample (Guo et al., 2014). The Pearson correlation analysis was conducted by SPSS software.

### Reference gene validation

The *miR319* and *miR393*, which have been reported to be responsive to cold stress in sugarcane and other plant species (Zhang et al., 2009; Thiebaut et al., 2012; Wang et al., 2014) and also detected in our high-throughput sequencing results (unpublished), were chosen as target genes to confirm the suitability of the candidates which were evaluated in this study. The expression of two target genes was calculated with the 2^−ΔΔ*Ct*^ method (Schmittgen and Livak, 2008; Luo et al., 2014).

## Author contributions

Conceived and designed the experiments: YY, LX, JG. Performed the experiments: YY, XZ, YC, SG. Analyzed the data: YY, HL. Wrote the paper: YY, LX, JG, YS. Revised the final version of the paper: LX, JG, YQ. Approved the final version of the paper: LX and YQ.

### Conflict of interest statement

The authors declare that the research was conducted in the absence of any commercial or financial relationships that could be construed as a potential conflict of interest. The reviewer, Dr. Xin Guan, and Handling Editor declared their shared affiliation, and the Handling Editor states that the process nevertheless met the standards of a fair and objective review.
