# Supplementary material for: Selection of Reference Genes for Normalization of MicroRNA Expression by RT-qPCR in Sugarcane Buds under Cold Stress
Source: Front Plant Sci. 2016 Feb 5;7:86. doi: 10.3389/fpls.2016.00086 (PMC4742636; doi:10.3389/fpls.2016.00086)
Supplement: Supplementary file 1 [file DataSheet1.doc]

Supplementary Material

# Selection of reference genes for normalization of microRNA expression by RT-qPCR in sugarcane buds under cold stress

Yuting Yang, Xu Zhang, Yun Chen, Jinlong Guo, Hui Ling, Shiwu Gao, Yachun Su, Youxiong Que, Liping Xu*

*** Correspondence:** Liping Xu: xlpmail@126.com

**Supplementary table**

**Supplementary Table 1. The mean Ct values of the 13 candidate reference genes in FN39 and ROC22 cultivars at various time points with cold treatment.**

| Cultivar | Stress | *miR159* | *miR160* | *miR167* | *miR171* | *miR398* | *miR1520* | *miR5059* | *miR5072* | *miR5655* | *5S*  *rRNA* | *18S rRNA* | *GAPDH* | *eEF-1α* |
| --- | --- | --- | --- | --- | --- | --- | --- | --- | --- | --- | --- | --- | --- | --- |
| FN39 | CK | 21.427 | 25.527 | 21.532 | 20.234 | 25.042 | 17.461 | 19.075 | 15.909 | 28.328 | 19.205 | 14.518 | 21.488 | 20.267 |
|  | Cold-1h | 20.198 | 24.623 | 21.902 | 20.368 | 25.041 | 18.145 | 18.567 | 15.911 | 28.266 | 19.699 | 14.84 | 22.571 | 20.795 |
|  | Cold-3h | 21.518 | 25.468 | 21.806 | 20.999 | 24.533 | 17.795 | 19.717 | 15.597 | 27.999 | 19.56 | 14.98 | 22.801 | 21.207 |
|  | Cold-6h | 21.109 | 24.947 | 21.209 | 20.08 | 24.443 | 15.244 | 19.277 | 16.389 | 28.188 | 18.847 | 14.536 | 21.959 | 20.247 |
|  | Cold-12h | 21.045 | 24.746 | 22.104 | 20.579 | 24.82 | 17.206 | 22.525 | 20.299 | 29.203 | 19.595 | 14.882 | 22.413 | 21.216 |
|  | Cold-24h | 20.149 | 24.145 | 21.668 | 21.244 | 23.803 | 17.058 | 18.803 | 15.542 | 27.331 | 19.57 | 14.924 | 23.116 | 21.353 |
|  | Cold-48h | 21.279 | 25.5015 | 21.9845 | 20.706 | 24.003 | 16.923 | 19.521 | 15.703 | 29.046 | 19.214 | 14.675 | 22.229 | 21.059 |
| ROC22 | CK | 20.691 | 25.811 | 21.371 | 20.428 | 25.007 | 17.71 | 19.7 | 16.202 | 27.952 | 18.085 | 14.121 | 20.732 | 19.201 |
|  | Cold-1h | 21.17 | 26.265 | 22.041 | 20.914 | 25.705 | 19.044 | 20.681 | 17.358 | 28.817 | 19.09 | 14.4 | 22.109 | 20.845 |
|  | Cold-3h | 19.255 | 24.69 | 20.171 | 19.399 | 23.968 | 17.157 | 18.631 | 15.365 | 26.939 | 18.068 | 14.183 | 21.212 | 19.516 |
|  | Cold-6h | 20.138 | 25.441 | 20.929 | 20.011 | 24.68 | 17.339 | 18.615 | 16.181 | 28.042 | 18.224 | 14.147 | 21.265 | 19.74 |
|  | Cold-12h | 20.385 | 25.573 | 21.1 | 20.35 | 25.031 | 17.693 | 18.698 | 15.89 | 27.994 | 18.522 | 14.243 | 21.831 | 20.264 |
|  | Cold-24h | 19.358 | 24.848 | 20.221 | 19.746 | 24.473 | 17.312 | 18.565 | 15.094 | 27.762 | 18.467 | 14.198 | 21.514 | 20.218 |
|  | Cold-48h | 20.432 | 25.621 | 21.136 | 20.337 | 25.146 | 18.159 | 20.065 | 16.048 | 28.201 | 19.018 | 14.435 | 21.903 | 20.49 |

**Supplementary figure**


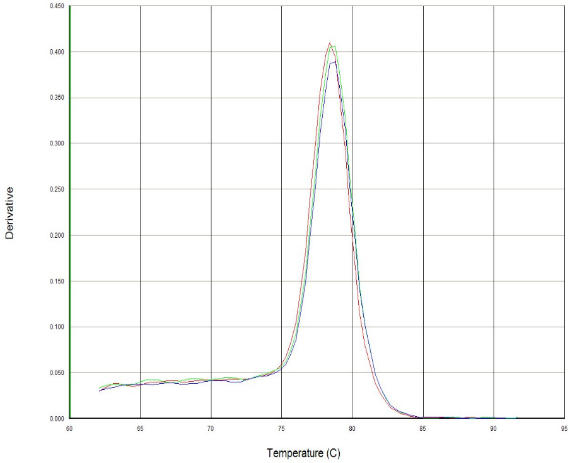

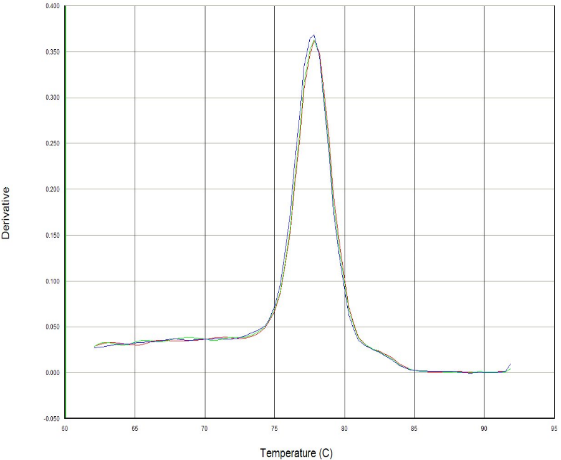

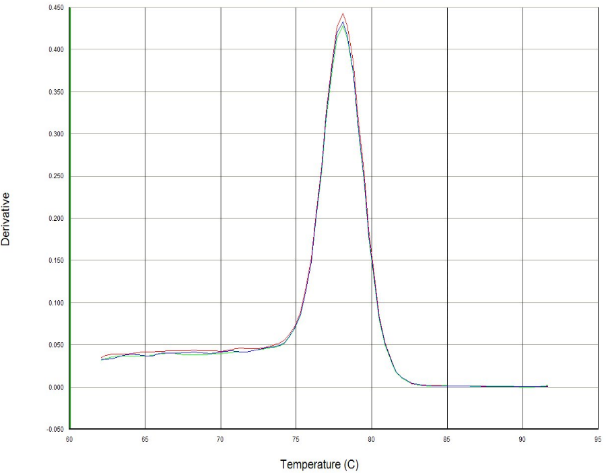

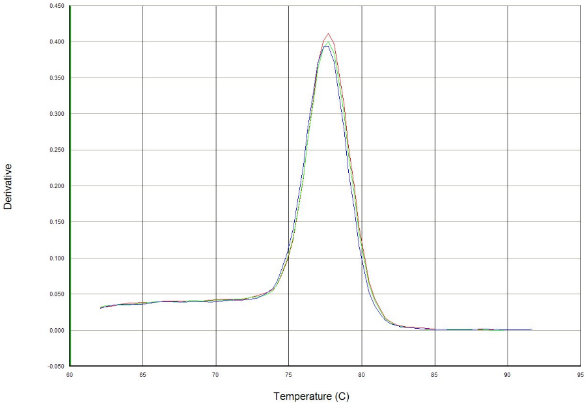

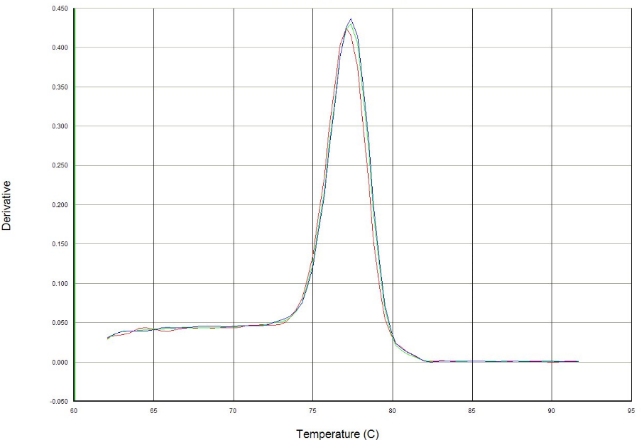

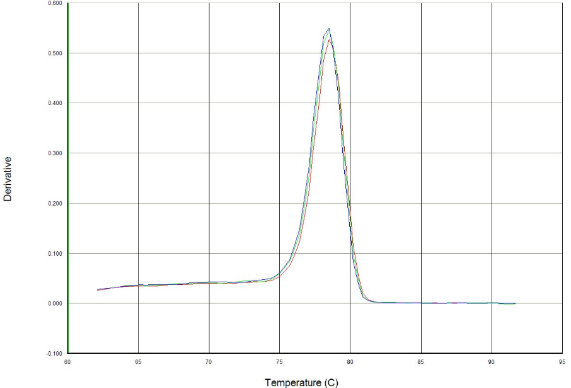

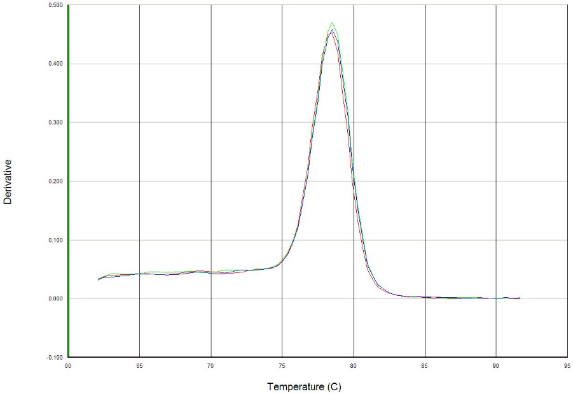

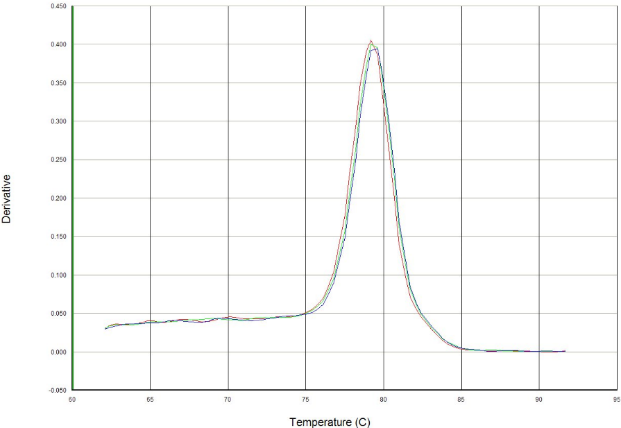

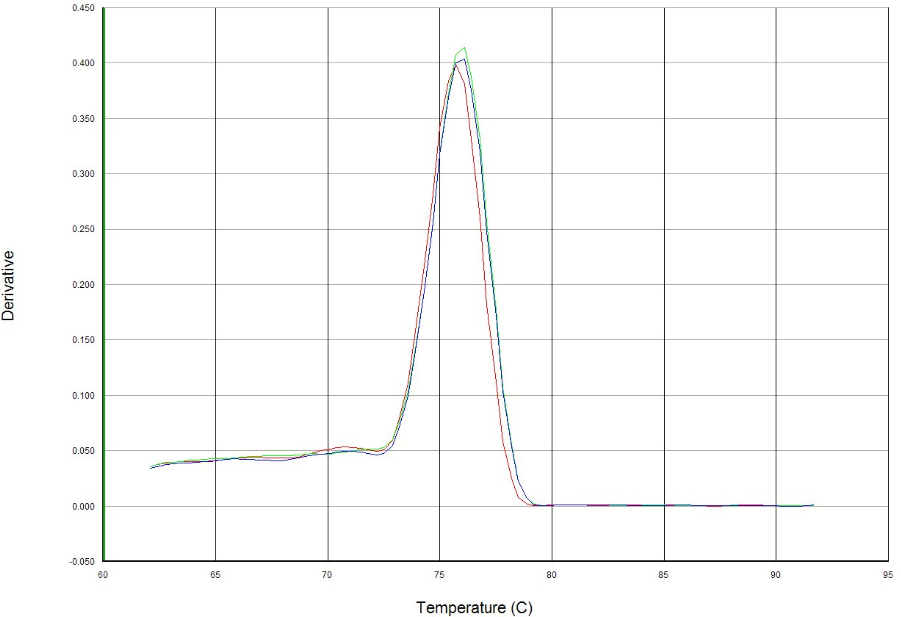

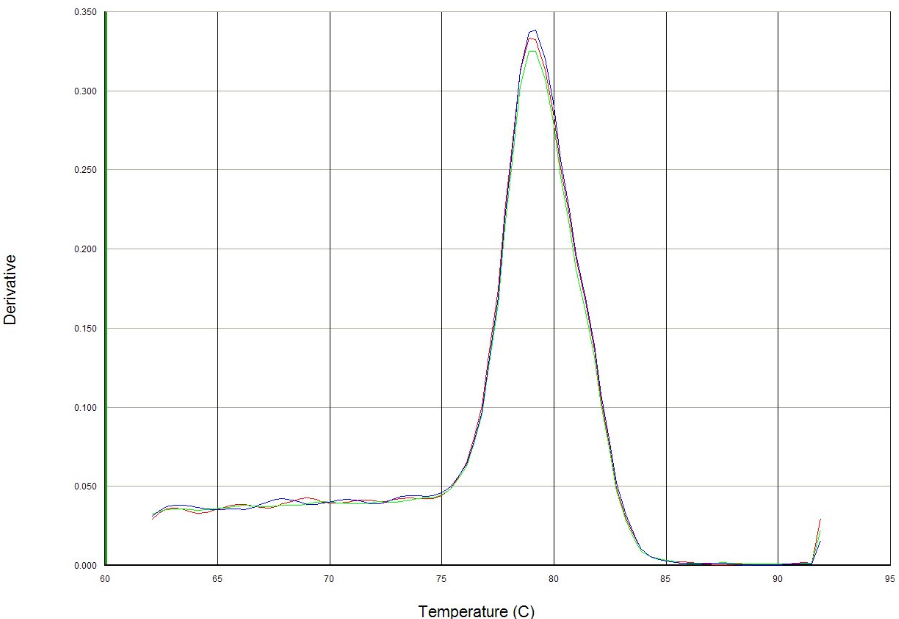

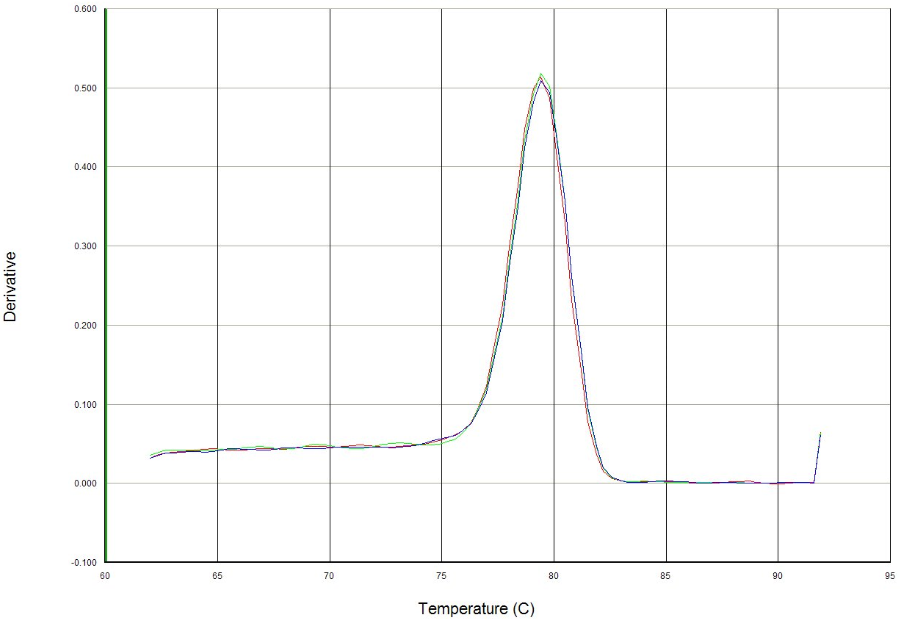

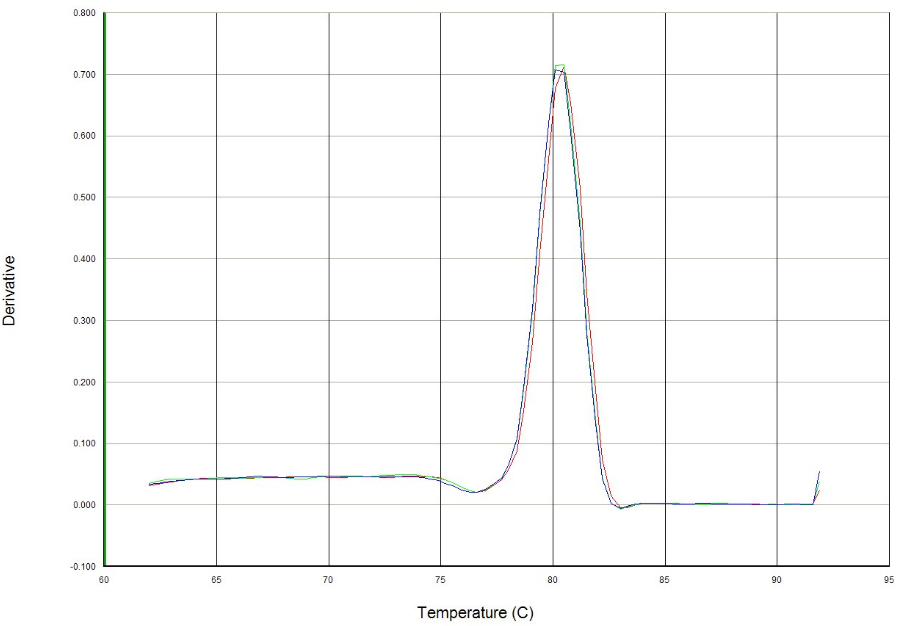

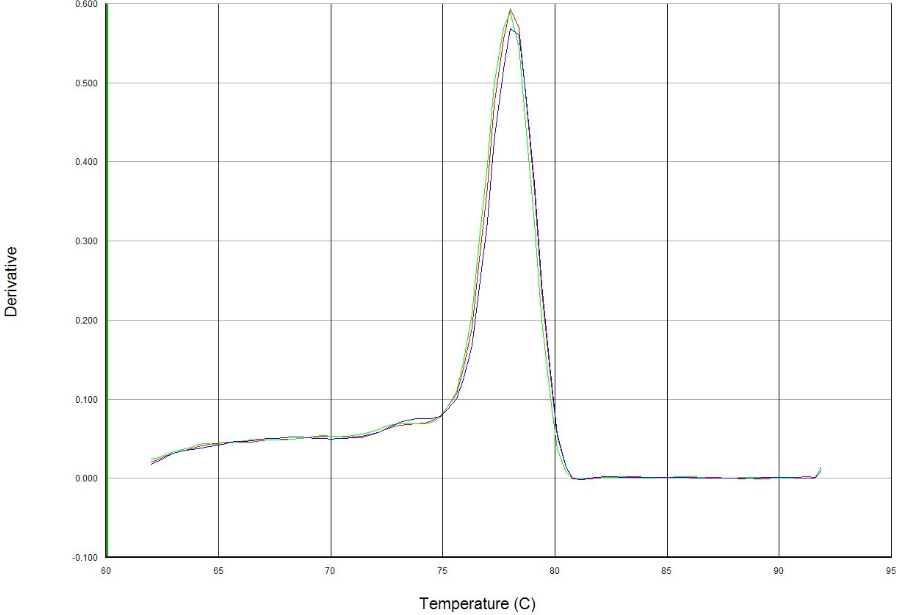

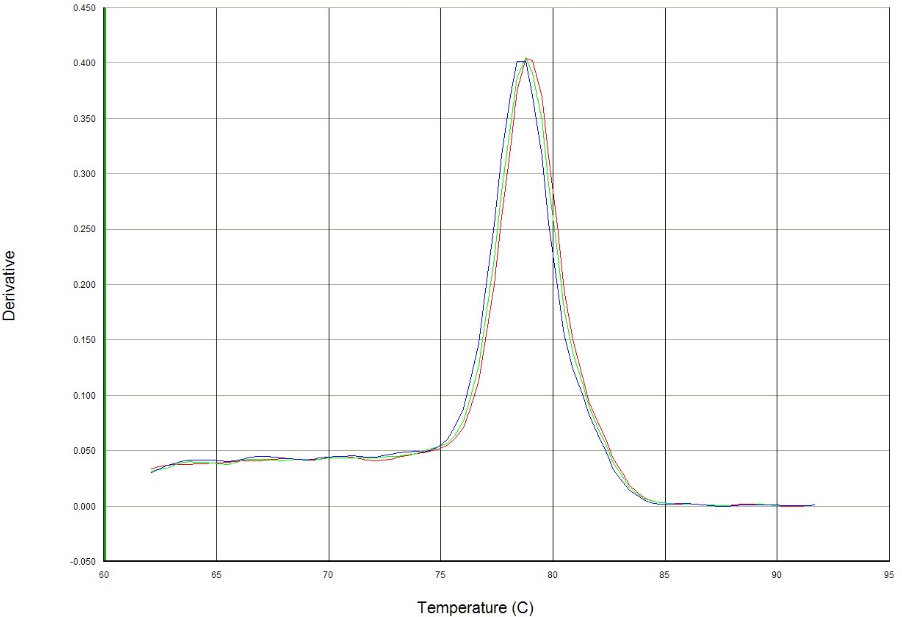

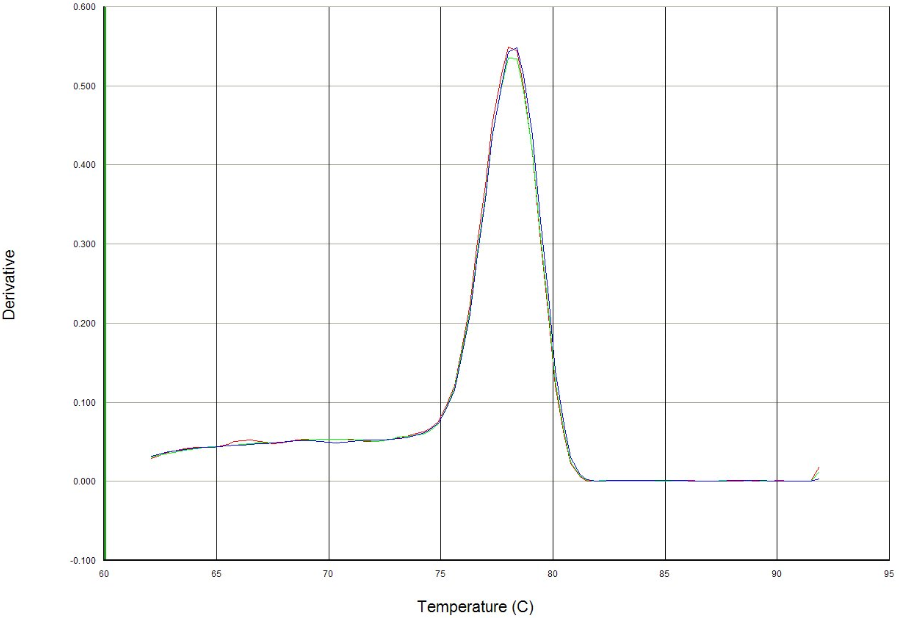


**A**

**B**

**C**

**D**

**E**

**F**

**G**

**H**

**I**

**N**

**M**

**L**

**K**

**G**

**O**

**Supplementary Figure 2.** **The melting curves of genes. The A-O represent *miR159*, *miR160*, *miR167*, *miR171*, *miR398*, *miR1520*, *miR5059*, *miR5072*, *miR5655*, *5S rRNA*, *18S rRNA*, *GAPDH*, *eEF-1α*, *miR319 and miR393*, respectively.**
